# Dentists' lived experience of providing dental care during the COVID-19 pandemic: A qualitative study in Mashhad, Iran

**DOI:** 10.3389/froh.2023.1095240

**Published:** 2023-03-01

**Authors:** Melika Hoseinzadeh, Zahra Sa’adAbadi, Sara Maleki Kambakhsh, Saber Babazadeh

**Affiliations:** ^1^ Student Research Committee, Faculty of Dentistry, Mashhad University of Medical Sciences, Mashhad, Iran; ^2^ Private Practitioner, Mashhad, Iran; ^3^ Dental Caries Prevention Research Center, Faculty of Dentistry, Qazvin University Medical Sciences, Qazvin, Iran; ^4^ Department of Community Oral Health, Faculty of Dentistry, Mashhad University of Medical Sciences, Mashhad, Iran

**Keywords:** COVID-19, dental care, qualitative research, dentists, SARS-CoV-2

## Abstract

**Background:**

This study aimed to explore the lived experiences of dentists practicing in Mashhad, Iran, during the COVID-19 pandemic and identify possible influencing factors for providing dental health in this era.

**Methods:**

Sixteen dentists took part in this qualitative phenomenological study. Semi-structured face-to-face interviews were performed and evaluated using a qualitative content analysis approach. The MAXQDA program (2020) was used to code and classify the data. Purposive sampling, audio recording, member checking, and peer review were employed to verify transferability, reliability, and validity. The interview transcripts were transcribed, and sentences and remarks relevant to the study's goal were retrieved and classified.

**Results:**

Six hundred twenty-eight codes were extracted after several readings of the texts. Five core themes and fourteen sub-themes were determined after deleting repeated themes and axial coding. The main five themes included: “Coping with the COVID-19 pandemic”, “Alternations in providing dental care,” “Infection control,” “COVID-19-related news and information sources”, and “Positive and negative aspects of COVID-19 in dental care ”.

**Conclusions:**

During the COVID-19 pandemic, dentists noted various challenges in delivering routine dental treatment, including a lack of resources, reliable and straightforward guidelines for dentists, and governmental policies for dental facility restrictions. Dentists concluded that financial, educational, and financial support from the government would significantly enhance the delivery of community dental care services. With the right strategies and lessons learned, dentists may be better prepared for future challenges in global health care.

## 1. Introduction

Since late December 2019, the severe acute respiratory syndrome coronavirus 2 (SARs-CoV-2) has spread worldwide, causing a global public health concern known as Coronavirus disease 2019 (COVID-19) (1, 2). The disease has affected millions of people globally. Iran ranked among the most COVID-19-affected nations, with over 7,5 million verified cases (3). In Iran and many other countries, public places, particularly dental offices and dental faculties, were temporarily closed to stop the spread of COVID-19 (4). However, dental care cannot be delayed for long periods of time due to the risk of severe complications (5). As a result, understanding the shortcomings of a dental care system is critical for overcoming existing oral health challenges during the COVID-19 pandemic and preparing for potential global challenges.

Dental professionals have been at risk of contracting several airborne infections, including COVID-19, due to their proximity to patients and bodily fluids and the production of significant levels of droplets and aerosols (3, 5–8). Several studies have been conducted on the actions taken in dental clinics to minimize infection transmission, with the most significant emphasis on infection control (5). The deficiencies and barriers in providing dental care, however, have been reported in the studies. A survey done in Poland found that a lack of advanced personal protective equipment (PPE) and poor coordination of health services related to the pandemic, and a deficit of PPE were to blame for dental professionals' fear and a drop in the number of dental procedures (9). In another study, increased treatment costs due to the increased use of protective measures resulted in lower patient attendance (10).

The psychological effects of COVID-19 have also negatively impacted dental professionals (11). Dental professionals have reported financial hardship due to reduced workload, restrictions on specific dental procedures, and high PPE costs (12, 13). More than half of the Iranian dentists had to spend their savings on daily expenses in June 2020 (13).

Because of the diversity in healthcare and governmental policies and the availability of resources in different countries, the experience of the COVID-19 phenomenon seems to vary significantly among healthcare professionals, making it critical to conduct local studies. Qualitative research aims to understand human behavior, perspectives, beliefs, and personality traits that are difficult to recognize in quantitative studies (14). Dentists' narratives on providing dental health care during the COVID-19 pandemic are critical for exposing their supportive and educational requirements during health crises. Therefore, this qualitative study aimed to investigate dentists' lived experiences delivering dental care during the COVID-19 pandemic in Mashhad, Iran, and identify relevant factors.

## 2. Material and methods

### 2.1. Design and setting

A qualitative phenomenological approach was used in this investigation. Phenomenology is the study of how lived experiences of phenomena contribute to the meaning of one's life (15). This present study was conducted between May 2020 and October 2021 in Mashhad, the second most populous city in Iran. During this time, the number of confirmed COVID-19 cases in Iran rose from nearly 92 thousand to 5.58 million, and almost 70% of people were vaccinated.

### 2.2. Sampling

Using purposive and snowball sampling methods, the Iranian dentists, who were all practicing dentistry during the COVID-19 pandemic at the time of our study, were selected to participate. Dentists practicing in private offices or private/public clinics in high-income and low-income districts were chosen to get the maximum variation. The districts of Mashhad were classified based on income level and social sustainability in a study by Mafi et al., “Assessment of Social sustainability in Mashhad Metropolis” (16).

### 2.3. Data collection

Semi-structured face-to-face interviews were conducted in Persian to collect data. Dental practices closed in March 2020 and reopened in May 2020; therefore, we could conduct all interviews in dentists’ offices. Interview sessions lasted between 45 and 60 min and were conducted by one researcher (ZS) using the interview guide (Table 1). The interviews were recorded to improve the reliability of the data. Permission to record the interview was requested, and all participants gave their written consent. The audios were carefully transcribed on the same day.

**Table 1:** The semi-structured interview protocol.

How are you coping with the COVID-19 pandemic?
How do you modify your approach to providing dental care to your patients?
How do you keep yourself safe when practicing during the pandemic?
How do you keep your patients safe while practicing?
What is your source of knowledge on the safety protocols?
What type of treatments do you offer throughout the pandemic?
What difficulties do you encounter when following the preventive protocols?
How have delivering dental services changed for you?
What is your financial situation like throughout the pandemic?
How does the dentistry community assist dentists during the pandemic?

The authors engaged in data analysis along with data collection. After the final interview, no new themes emerged. At this point, saturation had been achieved, meaning that no further information was provided to assist in comprehending the dentists' lived experience; hence no more participants were recruited (17, 18).

### 2.4. Data analysis

Two authors (SB and SM) independently conducted data analysis using MAXQDA 2020. Disagreements were resolved through conversation and all authors expressed their ideas about the themes and sub-themes, the process called “investigator triangulation”. Each transcript was coded and categorized following the seven steps of Colaizzi's analytic method as follows (19):

1) To ensure that the content of each transcript was understood, it was read several times.
2) The phrases and sentences that related to the research objective were identified.
3) The relevant statements were formulated in terms of their meaning.
4) For each interview, the previous steps were repeated, and the related meanings were categorized into themes.
5) Each theme was described in detail.
6) A detailed description of the objective phenomenon with a clear statement of its essential characteristics.
7) Discussing the fundamental statements with 5 participants to assess the credibility of the findings and their interpretations and improve the credibility of the results, a process called “member checking” (20).

In order to meet the transferability criteria, a detailed description of the themes and subthemes, along with participant quotes, was included in the results section.

### 2.5. Trustworthiness

Lincoln and Guba's criteria, including transferability, dependability, confirmability, and credibility, were followed to ensure the study's validity (18).

### 2.6. Ethical considerations

The protocol of this research was approved by the Research and Ethics Committee of Mashhad University of Medical Sciences (code: IR.MUMS.DENTISTRY.REC.1399.073). The goal of the study was properly explained to the participants, and their written consent was acquired. They were also informed that their identities would be kept private and that they could resign from the research at any time.

## 3. Results

A total number of 16 dentists were interviewed. Table 2 displays the demographic characteristics of the participants.

**Table 2:** The participants’ demographic characteristics.

Demographic characteristics	Subgroups	Number (%)
Gender	Female	8 (50)
	Male	8 (50)
	Total	16 (100)
Age range (years)	20–40	9 (56.25)
	40–60	7 (43.75%)
	Total	16 (100)
Specialization	General dentist	9 (56.25)
	Specialists	7 (43.75%)
	Total	16 (100)
Type of practice	Private office^a^	6 (37.5%)
	Public clinic	6 (37.5)
	Semi-private clinic^b^	2 (12.5)
	Private clinic^c^	2 (12.5)
	Total	16 (100)
Clinical experience (years)	1–5	3 (18.75)
	5–10	6 (37.5)
	10–15	2 (12.5)
	15–20	3 (18.75)
	>20	2 (12.5)
	Total	16 (100)
District	High-income	11 (68.75)
	Low-income	5 (31.25)
	Total	16 (100)

^a^ In private offices one or two dentists practice dentistry.

^b^ Semi-private clinics are clinics where in the mornings, they have governmental staff and established governmental tariffs, and in the afternoons, the tariffs become the same as private sections with different staff.

^c^ Private clinics are different from private offices because more dentists practice in one clinic.

**Figure 1:**
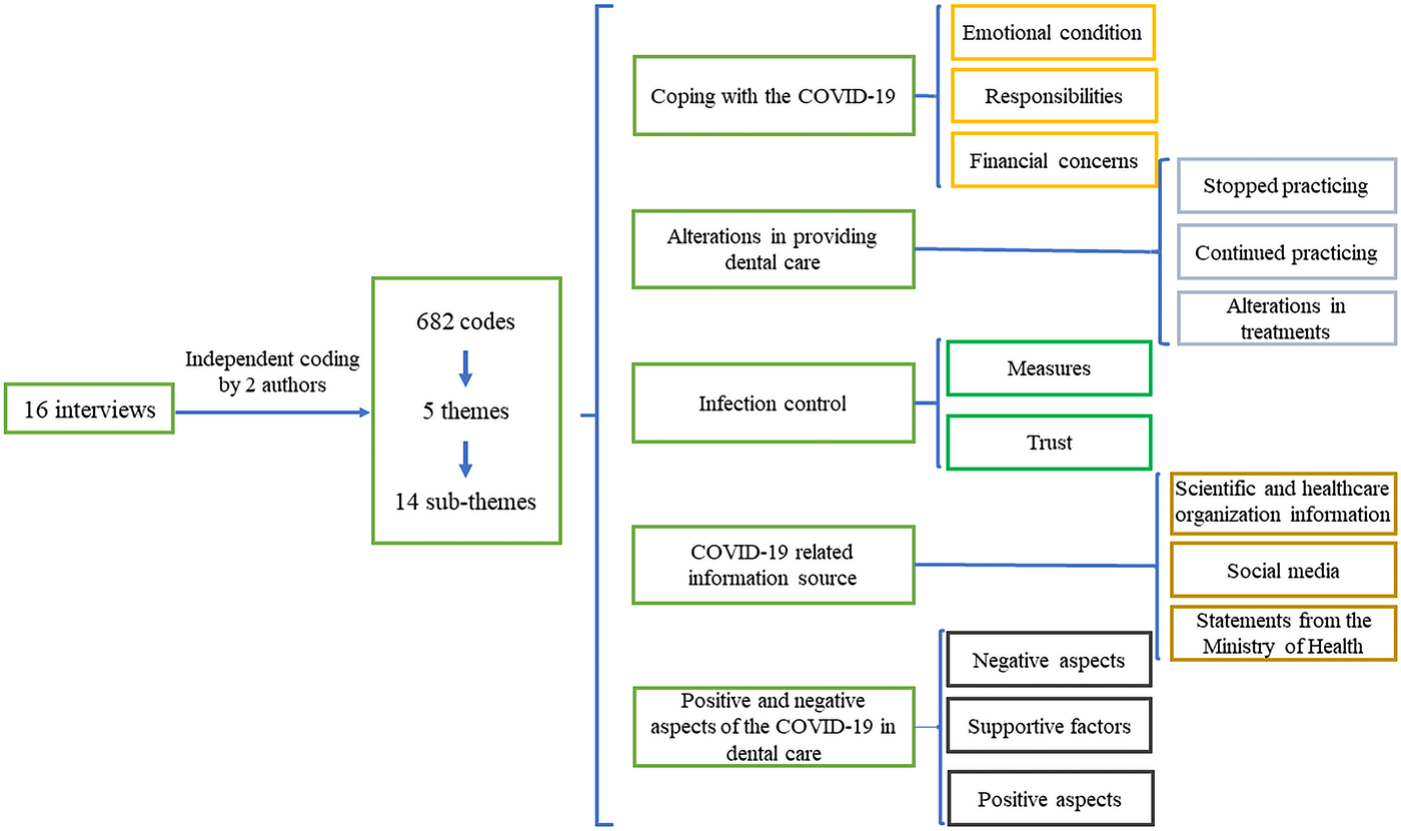
The study flow diagram containing the main themes and sub-themes.

From the interviews with dentists, 682 initial codes, five themes, and fourteen sub-themes emerged (Figure 1). The main themes included the following:

### 3.1. Coping with the COVID-19 pandemic

#### 3.1.1. Emotional condition

Most dentists reported that the feelings of fear, stress, despair, and anxiety during the COVID-19 pandemic affected their mental health. The most common reasons were insufficient information about the disease, fear of sickness and death, and the ambiguity of the future of their practice. One dentist commented:

“Well, since it was something unknown, (our) stress was at the maximum level. I mean, we felt our lives would change upside down, and we couldn’t practice like in the past. We wondered how the future would become. Whether we could practice like the past or not”.

#### 3.1.2. Responsibilities

Some of the participants mentioned the responsibility they felt. They believed that following the protocols could impede disease transmission; hence they continued to practice. However, they mentioned that practicing while adhering to the guidelines and wearing PPE was exhausting.

#### 3.1.3. Financial concerns

The participants also mentioned the pandemic's effects on their personal lives. Most dentists reported their financial situation was worsening because of high PPE costs, decreased patient attendance, decreased working hours, quarantine, and patients' financial hardship. One of the dentists reported that patients had visited mostly for emergency treatments, not for “more profitable cosmetics services and crowns”. Few dentists reported that a decrease in the number of practicing dentists increased their number of patients, and improved their financial situation.

### 3.2. Alterations in providing dental care

#### 3.2.1. Stopped practicing

Many dental clinics shut down or limited their working hours, especially at the beginning of the pandemic. The causes included fear of sickness, not having a thorough knowledge of the disease, and the general lockdown at the beginning of the pandemic.

#### 3.2.2. Continued to practice

Nonetheless, several dentists in this research continued to practice because of a sense of responsibility, care for their patients, and a desire to offer dental services and handle incomplete and emergency treatments. Some dentists had even extended their hours because of increased patient numbers when many dentists stopped practicing. One of the dentists who remained to practice stated: “I really felt that this was our responsibility. It wasn”t ethically correct to stop treating patients at that time just to keep ourselves safe. Imagine that if my brother, my sister, my friend, and I had a toothache, what would we do? Someone must have been there to provide the treatment”.

#### 3.2.3. Alterations in treatments

Aside from changes in working hours, alterations in the types of provided dental treatments and treatment methods also happened. Most dentists agreed that they treated the emergency cases like “alleviating pain, completing patients RCT and extractions” and postponed the elective services “like restorations, cosmetic services, or SRP”. In some cases, some minimal modifications were made in the treatment methods to protect patients and their health. One dentist noted:

“We minimally modified some of the treatments that generated aerosols. For example, for inserting implants, we need lots of irrigation. We manually used water syringes and slowed down our devices to reduce aerosol generation. However, the overall principles were the same”.

### 3.3. Infection control

#### 3.3.1. Measures

Dentists talked about their workplace arrangements and using PPE. Patient-related infection control was also discussed. Dentists mentioned screening patients with pulse oximetry, temperature check, and asking for PCR test results. Many said that patients' companions were not allowed in the waiting room, and they were asked to maintain social distancing. Several dentists provided masks, gloves, or disinfectant solutions for the patients.

Numerous respondents reported using air conditioning and rubber dam and providing protective eyeglasses and antimicrobial mouthwash for patients. Most dentists reported sterilization and disinfection of equipment and the environment.

#### 3.3.2. Trust

Adhering to the health guidelines also was a way to gain patients' trust as people had become more sensitive to sanitation, as some dentists mentioned. One dentist said:

“When I inform my patients that I work in a less crowded clinic, they would come with pleasure, even if they had to pay more. But it's not for all patients. Well, patients with higher (socioeconomic) status would do that”.

### 3.4. COVID-19-related information source

#### 3.4.1. Scientific and healthcare organization information

Many dentists read the relevant information on the internet, websites, and articles. According to one dentist, he used the World Health Organization (WHO) and the Centers for Disease Control and Prevention (CDC) websites, as well as studies from scientific journals, to “use better, more accessible, and accurate guidelines”.

#### 3.4.2. Social Media

Other dentists relied on TV and social media. One dentist noted, “There was general information on TV and social media. For example, one academic practitioner explained air conditioning, and we conducted the same”.

#### 3.4.3. Statements from the Ministry of Health

Some dentists found the statements useful, while others complained about a delay compared to the WHO website and a lack of specialized declarations for dentists. According to one participant, “the Ministry of Health supplied national summaries of CDC and WHO recommendations”, and he advised checking the global guidelines first. Many dentists recommended that governmental guidelines about protection protocols and PPEs would decrease the uncertainties and concerns about infection control principles.

### 3.5. Positive and negative aspects of COVID-19 in dental care

#### 3.5.1. Negative aspects

Most of the challenges stemmed from the high cost and shortage of PPEs, particularly during the start of the pandemic. Few dentists reported a severe treatment burden, as many discontinued practicing. According to one dentist: “We called the COVID-19 era “the era without endodontists”. No endodontist was willing to work. I'm good at endodontics, but not every tooth. Or for example, it's been said that pediatric patients were ticking bombs because they didn't have symptoms and were silent carriers. No pediatrics worked in that era, either. So, we treated children too”.

Many dentists recommended that dental facilities during pandemics would be better stay open to prevent increasing treatment overloads and delays in treatments. The closing of Mashhad's Faculty of Dentistry clinic added to the workload of other facilities. One dentist commented: “At that time, we had a lot of patients with strange oral lesions. I had to deal with many strange oral lesions myself because no colleagues in the university were there to consult”.

According to some dentists, their workplaces did not provide enough PPE for dentists and their assistants. Wearing PPE has also been reported to impact the quality of dentists' practice. One of the dentists stated that before the pandemic, he would do endodontic treatments or retreatments on every tooth, but that the working conditions had become so difficult that he treated fewer and more selective cases.

Some dentists reported that the clinics did not adhere to hygiene protocols. One dentist noted: “The gowns we use are disposable, and after you wear them, you have to throw them away. However, some clinics autoclaved the gowns for the next dentist, which was wrong”. Some dentists also reported that in many private offices, it was possible to keep patient intervals; however, in many clinics, especially public clinics, it had not complied. Therefore, many dentists recommended that surveilling dental clinics during healthcare crises like the COVID-19 pandemic would be necessary to ensure health protocol adherence.

Some dentists also stated that delayed treatments due to patients' fear of infection resulted in more complicated and expansive treatments, especially at the beginning of the pandemic. One dentist noted: “We are now seeing the effects of neglecting dental treatments during the pandemic's first year. Dental treatments were wrongly assumed to be very dangerous. I see a patient who has not visited a dentist for a year because of Corona comes, and when I look at his previous radiograph, many teeth that could have been restored now have become hopeless. This happened because of the fear of going to the dentist”.

#### 3.5.2. Supportive factors

Most dentists reported not receiving any support from the government or their workplaces. Some claimed that as government support, healthcare workers were given hand sanitizer and PPE coupons and were vaccinated sooner than the general public; yet, they claimed that there was no difference between dentists who were working and those who weren't. One dentist said: “I remember that medical system organization offered some N95 facemasks and sanitizer, which was beneficial because it was scarce back then. You could use your medical system organization card, pay, and receive the goods. However, there was no preference given to those who were practicing during the pandemic”.

Some dentists reported that their workplace did not provide any kind of support, especially providing PPE: “the clinic where I work gave us ordinary 3-layer surgical masks, even though N95 masks are relatively cheap, let al.one shields and gowns”.

Dentists stated that more assistance may have made providing oral health simpler, such as providing more PPE at reduced prices, protocols customized to the dental profession, and monitoring dental facilities for adherence to hygiene protocols.

#### 3.5.3. Positive aspects

Some positive aspects of the COVID-19 pandemic were also mentioned, including increased leisure time, improved knowledge by participating in online webinars, personal studies, and consulting colleagues about the COVID-19 situation. Some also mentioned that COVID-19 reminded them, especially about the infection control principles, as one dentist noted: “Corona was an excellent reminder for me to emphasize infection control, wearing shields, etc”.

## 4. Discussion

The purpose of this study was to gain a better understanding of the perspectives of dentists practicing in Mashhad during the COVID-19 pandemic. One year after one of the most disastrous pandemics, dental professionals' unique experiences may give insight into the profession's coping mechanisms, the healthcare system's shortcomings, and lessons for potential future challenges. Our findings highlighted some significant elements of dentists' experiences during the pandemic as healthcare workers who are susceptible to infection and have physically demanding jobs. Based on the researchers' current knowledge, this was the first qualitative study examining dentists' lived experiences during the COVID-19 pandemic in Iran.

Many dentists in our study confirmed that they refused to practice during the COVID-19 pandemic due to anxiety about the unknown disease, particularly in the early stages of the outbreak. Noushi et al. and Miśta et al. also observed that many dental clinics closed due to insufficient personal protective equipment and subjective assumptions regarding the spread of the disease (9, 21). According to a survey in the United States in 2020, COVID-19 prevalence was 0.9% among dentists (3). Another research in Canada in 2020–2021 revealed the infection rate among dentists was as low as 1.08% (22). It may be the result of the fact that infection control principles have always been an inherent aspect of dental practice. Our study was conducted one year after the COVID-19 pandemic onset, and most participants stated that by adhering to health and safety measures, they could continue to practice as previously, and access to PPE was also improved during this time. Similarly, Tysic-Mita et al. found that the availability of PPE was a significant determining factor for Polish dentists in selecting whether to continue working throughout the pandemic (9). However, in our study, most dentists reported a shortage of PPE and received no support from the government or their workplace during the early phases of the pandemic. Similarly, Ahmadi et al. reported that 87% of Iranian dentists in their study had problems providing PPE during the pandemic (13). In another qualitative study in Tehran, the capital of Iran, the participants with high-risk occupations reported that difficulty accessing health supplies was among the structural factors of not following COVID-19 health instructions (23).

Fear, worry, tension, and despair were the most common sentiments among dentists in our study. Compared to the general population, healthcare professionals encountered far more emotional stress during the COVID-19 pandemic (24, 25). Similarly, Noushi et al. discovered that Canadian dentists are worried about their employees, patients, personal health, and the future of their practice (21). Fear, worry, and perplexity were also noted by Mita et al. among Polish dentists (9). A recent meta-analysis of dentists' mental health found that they had a high degree of COVID-19 anxiety, and considerable levels of depression, worry, and rage (26). Fatigue and physical tiredness were also described as side effects of the pandemic and the psychological impact (26). For health professionals, including dentists, psychological support is as crucial as physical safety, especially during the COVID-19 pandemic (27). Ahmadi et al. reported that about half of the participant dentists reported the symptoms of depression and anxiety and the need to consult with a psychiatrist or a therapist (13). Our findings, consistent with the literature, imply that mental support may aid dentists' well-being and oral healthcare service quality, particularly during challenging times.

Some dentists in our study reported continuing to practice as they felt responsible for the patient. Likewise, Tysic-Mita et al. claimed that the major motive for the Polish dentists who maintained their professional activity throughout the pandemic was the altruistic necessity to give emergency and urgent dental procedures (9).

Almost all dentists in our study acknowledged using PPE and organizing patient receptions to protect patients and gain their trust. In Aquilanti et al. study, the high level of people's trust in their dentists was related to the trust in implemented infection control measures (28). Our participants also made some changes in the provided treatments and methods, as it was recommended that aerosol-generating procedures should be avoided (29). Ahmadi et al. also reported that 70% of their participants only provided emergency treatments during the pandemic (13). Similarly, Tarakji et al. and Carvalho et al. reported that most dentists in their studies followed the protective guidelines (30, 31). According to Wu et al., hydrogen peroxide, rubber dams, ultrasonic tools, and panoramic radiographs should be used instead of intraoral radiographs. They also suggested extending the interval between visits to maintain social distancing (32). Similarly, most participants in our study reported that they did not allow persons accompanying patients in the waiting room to avoid crowding.

Many participants reported using websites and social media as their primary sources of knowledge. Likewise, Duruk et al. reported that 96.27% of Turkish dentists acquired knowledge of COVID-19 through personal blogs, medical experts, and social media (33). Unlike our findings, De Stefani et al. reported that 70% of the dentists obtained COVID-19-related knowledge from expert physicians, National Health Institute, or expert dentists, 49% used social networks, and only 7% used scientific literature (34). The differences might be associated with the dentists' perceived quality of the available information sources. n our study, some dentists were pleased with the Ministry of Health's pronouncements, while others were not. Dissatisfaction was exacerbated by the lack of specific guidelines for dentists and the delay in reporting. Dental care quality would have been enhanced if dentists had received accurate and timely information. Similarly, Al-Amer et al., in their qualitative study on Jordanian dentists, reported that lack of clear guidelines was among the factors that led to mental distress (35).

The majority of the dentists in the current study claimed that a decrease in working hours and patient attendance, working environment, and insufficient governmental support led to their financial hardship. Similar research has found that the pandemic has had a detrimental impact on dentists' financial situation (36, 37). Ahmadi et al. also reported 97% of the dentists encountered a decrease in their financial income, and most of them reduced their working hours (13). Moreover, Al-Amer et al. reported that insufficient government support made sustaining infection control principles difficult and created financial hardship (35).

There were a few limitations to this study. Since dental settings were selected as interview places due to the COVID-19 situation, obtaining the cooperation of dentists and dental clinic managers was challenging. Moreover, voluntary participation excluded perceptions of teachers who did not wish to participate and may have had different views to share. However, sampling was conducted from diverse city districts, various treatment facilities, and up to the actual saturation limit to maximize the diversity of acquired data.

The lesson learned from the experience of the COVID-19 pandemic from the current study in case of a future healthcare crisis includes the implementation of specific protective protocols for dental professions as soon as possible, not restricting dental services, speeding up immunizations, and monitoring dental centers. Moreover, offering enough PPE at reduced, and financial support, for example in terms of low-interest loans may be highly advantageous in providing dental health.

## 5. Conclusion

Dentists identified many barriers to providing oral treatment during the COVID-19 outbreak. The majority of participants underlined the significance of government and dental society's practical support and knowledge during times such as the pandemic in order to improve oral health care delivery. Dentists have reported that governmental dental services were shut down, particularly at the start of the pandemic, due to a lack of awareness, fear of sickness and disease spreading, and inaccessibility to PPE and basic protective practices. The findings of this study will be used to improve dental services during the current pandemic and possible future healthcare crises by supporting the physical and mental health of dentists by making suitable arrangements.

## Data Availability

The datasets presented in this article are not readily available because the datasets contain participants interview full text that may reveal their identities. Requests to access the datasets should be directed to babazadehs@mums.ac.ir.

## References

[1] Meng L Hua F Bian Z. Coronavirus disease 2019 (COVID-19): emerging and future challenges for dental and oral medicine. J Dent Res. (2020) 99:481–7. 10.1177/0022034520914246 32162995 PMC7140973

[2] Li Q Guan X Wu P Wang X Zhou L Tong Y Early transmission dynamics in Wuhan, China, of novel coronavirus–infected pneumonia. N Engl J Med. (2020) 382:1199–207. 10.1056/NEJMoa2001316 PMC7121484 31995857

[3] Araujo MWB Estrich CG Mikkelsen M Morrissey R Harrison B Geisinger ML COVID-19 among dentists in the United States: a 6-month longitudinal report of accumulative prevalence and incidence. J Am Dent Assoc. (2021) 152:425–33. 10.1016/j.adaj.2021.03.021 34044974 PMC8142320

[4] Parvaie P Osmani F. Dentistry during COVID-19: patients’ knowledge and satisfaction toward health protocols COVID-19 during dental treatment. Eur J Med Res. (2022) 27:3. 10.1186/s40001-021-00629-0 35016707 PMC8749922

[5] Brunello G Gurzawska-Comis K Becker K Becker J Sivolella S Schwarz F Dental care during COVID-19 pandemic: follow-up survey of experts’ opinion. Clin Oral Implants Res. (2021) 32(Suppl 21):342–52. 10.1111/clr.13783 34196051 PMC8444799

[6] Attia S Howaldt H-P. Impact of COVID-19 on the dental community: part I before vaccine (BV). J Clin Med. (2021) 10:288. 10.3390/jcm10020288 33466777 PMC7830640

[7] Becker K Brunello G Gurzawska-Comis K Becker J Sivolella S Schwarz F Dental care during COVID-19 pandemic: survey of experts’ opinion. Clin Oral Implants Res. (2020) 31:1253–60. 10.1111/clr.13676 33047356 PMC7675432

[8] Chen L Zhao J Peng J Li X Deng X Geng Z Detection of SARS-CoV-2 in saliva and characterization of oral symptoms in COVID-19 patients. Cell Prolif. (2020) 53:e12923. 10.1111/cpr.12923 33073910 PMC7645955

[9] Tysiąc-Miśta M Dziedzic A. The attitudes and professional approaches of dental practitioners during the COVID-19 outbreak in Poland: a cross-sectional survey. Int J Environ Res Public Health. (2020) 17:4703. 10.3390/ijerph17134703 32629915 PMC7370196

[10] Salgarello S Audino E Bertoletti P Salvadori M Garo ML. Dental Patients’ perspective on COVID-19: a systematic review. Encycloped. (2022) 2:365–82. 10.3390/encyclopedia2010022

[11] Consolo U Bellini P Bencivenni D Iani C Checchi V. Epidemiological aspects and psychological reactions to COVID-19 of dental practitioners in the northern Italy districts of Modena and reggio Emilia. Int J Environ Res Public Health. (2020) 17:3459. 10.3390/ijerph17103459 32429193 PMC7277877

[12] Askar H Krois J Göstemeyer G Schwendicke F. Secondary caries risk of different adhesive strategies and restorative materials in permanent teeth: systematic review and network meta-analysis. J Dent. (2021) 104:103541. 10.1016/j.jdent.2020.103541 33259888

[13] Ahmadi H Ebrahimi A Ghorbani F. The impact of COVID-19 pandemic on dental practice in Iran: a questionnaire-based report. BMC Oral Health. (2020) 20:1–9. 10.1186/s12903-020-01341-x PMC7711254 33272261

[14] Chai HH Gao SS Chen KJ Duangthip D Lo ECM Chu CH. A concise review on qualitative research in dentistry. Int J Environ Res Public Health. (2021) 18:942. 10.3390/ijerph18030942 33499023 PMC7908600

[15] Hall E Chai W Albrecht JA. A qualitative phenomenological exploration of teachers’ experience with nutrition education. AJHP. (2016) 47:136–48. 10.1080/19325037.2016.1157532 PMC4867867 27226814

[16] Mafi E Abdoulahzadeh M. Assessment of social sustainability in mashhad metropolis. J Urban Ecol Res. (2017) 8:65–78. https://dorl.net/dor/20.1001.1.25383930.1396.8.15.4.5

[17] Fusch PI Ness LR. Are we there yet? Data saturation in qualitative research. Qual Rep. (2015) 20:1408. 10.46743/2160-3715/2015.2281

[18] Merriam SB. Qualitative research: A guide to design and implementation. San Francisco, California: John Wiley & Sons (2009).

[19] Wirihana L Welch A Williamson M Christensen M Bakon S Craft J. Using colaizzi’s method of data analysis to explore the experiences of nurse academics teaching on satellite campuses. Nurse Res. (2018) 25:30. 10.7748/nr.2018.e1516 29546965

[20] Lincoln YS Guba EG. Criteria for Assessing Naturalistic Inquiries as Reports. (1988).

[21] Noushi N Oladega A Glogauer M Chvartszaid D Bedos C Allison P. Dentists’ experiences and dental care in the COVID-19 pandemic: insights from Nova Scotia, Canada. J Can Dent Assoc. (2021) 87:1488–2159. https://jcda.ca/l5 34343068

[22] Madathil S Siqueira WL Marin LM Sanaulla FB Faraj N Quiñonez CR The incidence of COVID-19 among dentists practicing in the community in Canada: a prospective cohort study over a 6-month period. J Am Dent Assoc. (2022) 153:450–9.e1. 10.1016/j.adaj.2021.10.006 35241268 PMC8565357

[23] SoleimanvandiAzar N Irandoost SF Ahmadi S Xosravi T Ranjbar H Mansourian M Explaining the reasons for not maintaining the health guidelines to prevent COVID-19 in high-risk jobs: a qualitative study in Iran. BMC Public Health. (2021) 21:848. 10.1186/s12889-021-10889-4 33941149 PMC8090924

[24] Greenberg AL Greenberg H. UK's response to COVID-19: crude, unadjusted mortality figures are not the whole story. Br Med J. (2020) 369:m2453. 10.1136/bmj.m2453 32561514

[25] Tan BYQ Chew NWS Lee GKH Jing M Goh Y Yeo LLL Psychological impact of the COVID-19 pandemic on health care workers in Singapore. Ann Intern Med. (2020) 173:317–20. 10.7326/M20-1083 32251513 PMC7143149

[26] Salehiniya H Hatamian S Abbaszadeh H. Mental health status of dentists during COVID-19 pandemic: a systematic review and meta-analysis. Health Sci Rep. (2022) 5:e617. 10.1002/hsr2.617 35509394 PMC9059210

[27] Owen C Seddon C Clarke K Bysouth T Johnson D. The impact of the COVID-19 pandemic on the mental health of dentists in Wales. Br Dent J. (2022) 232:44–54. 10.1038/s41415-021-3756-7 35031746 PMC8758985

[28] Aquilanti L Gallegati S Temperini V Ferrante L Skrami E Procaccini M Italian Response to coronavirus pandemic in dental care access: the DeCADE study. Int J Environ Res Public Health. (2020) 17:6977. 10.3390/ijerph17196977 32987661 PMC7579054

[29] Tarakji B Nassani MZ Alali FM Abuderman AA. COVID-19 Guidelines to protect healthcare workers at hospitals and dental professionals at dental office. Ethiop J Health Sci. (2020) 30:1037–42. 10.4314/ejhs.v30i6.23 33883850 PMC8047227

[30] Tarakji B Nassani MZ Alali FM ABA Alsalhani AB Alqhtani NR Bin Nabhan A COVID-19-Awareness and practice of dentists in Saudi Arabia. Int J Environ Res Public Health. (2021) 18:330. 10.3390/ijerph18010330 PMC7795178 33466301

[31] Carvalho JC Declerck D Jacquet W Bottenberg P. Dentist related factors associated with implementation of COVID-19 protective measures: a national survey. Int J Environ Res Public Health. (2021) 18:8381. 10.3390/ijerph18168381 PMC8391182 34444131

[32] Wu KY Wu DT Nguyen TT Tran SD. COVID-19's impact on private practice and academic dentistry in North America. Oral Dis. (2021) 27(Suppl 3):684–7. 10.1111/odi.13444 32472974 PMC7300727

[33] Duruk G Gümüşboğa Z Çolak C. Investigation of Turkish dentists’ clinical attitudes and behaviors towards the COVID-19 pandemic: a survey study. Braz Oral Res. (2020) 34:e054. 10.1590/1807-3107bor-2020.vol34.0054 32490887

[34] De Stefani A Bruno G Mutinelli S Gracco A. COVID-19 Outbreak perception in Italian dentists. Int J Environ Res Public Health. (2020) 17:3867. 10.3390/ijerph17113867 32485959 PMC7312651

[35] Al-Amer R Ramjan LM Maneze D Al-Rashdan O Villarosa AR Salamonson Y The impact of a pandemic on dental professionals’ work and personal lives: a qualitative study with implications for primary healthcare workers. Front Public Health. (2022) 10:963410. 10.3389/fpubh.2022.963410 36117606 PMC9477186

[36] Mekhemar M Attia S Dörfer C Conrad J. The psychological impact of the COVID-19 pandemic on dentists in Germany. J Clin Med. (2021):10:1008. 10.3390/jcm10051008 33801333 PMC7958334

[37] Vergara-Buenaventura A Chavez-Tuñon M Castro-Ruiz C. The mental health consequences of coronavirus disease 2019 pandemic in dentistry. Disaster Med Public Health Prep. (2020) 14:e31–e4. 10.1017/dmp.2020.190 32498741 PMC7300188

